# Immunohistochemistry as a diagnostic tool in pathology

**DOI:** 10.6026/973206300213595

**Published:** 2025-10-31

**Authors:** Divya Jayagopal, Kantharaju K.H, Himanshu Shekhar, Akita Gopinath, Pukur I. Thekdi, Pushya K. Babu

**Affiliations:** 1 Department of Pathology, ACS Medical College, Velappanchavadi, Chennai, Tamil Nadu, India; 2 Department of Pathology, FAME (Farookh Academy of Medical Education), Near Ilavala, Hunsur Taluk, Mysore, India; 3 Department of General Medicine, Medinirai Medical College and Hospital, Medininagar, Jharkhand, India; 4 Department of General Medicine, Sree Balaji Medical College Hospital, Chromepet, Chennai-44, India; 5 Department of General Surgery, Dr. N. D. Desai Faculty of Medical Science and Research, Dharmsinh Desai University College Road, Nadiad, Kheda, Gujarat, India; 6 Department of Pathology, JSS medical college, Bannimantap A Layout, Bannimantap, Mysuru, Karnataka, India

**Keywords:** Immunohistochemistry, diagnostic pathology, tumor markers, monoclonal antibodies, tissue staining

## Abstract

Immunohistochemistry (IHC) is an important tool in pathology and allows visualization of antigens when obtaining tissue specimens from patients and employs labeled antibodies directed against those antigens. IHC is helpful to improve tumor classification, identify pathogens and discover prognostic markers when distinguishing features are absent or minimized by morphological assessment. As routine IHC has emerged over the years, monoclonal antibodies and further development of automated stainers allow for increased sensitivity and specificity of antigens assessed by IHC. Since cancer classification relies upon IHC to subtype cancers, define the origin of metastases and to administer targeted therapy, IHC remains a valuable and necessary process for clinical pathology. Innovations continue to be made in IHC, such as new biomarkers, multiplexing and fluorescent technology.

## Background:

Immunohistochemistry (IHC) has been an important part of modern diagnostic pathology and is a significant step forward from the developments that began in the late 1940s [1]. While originally described for research applications, IHC has evolved into a standard clinical routine to explore specific cellular antigens in tissue sections through the visualization of antigen-antibody relationships through a variety of labelling systems [2]. The advents of monoclonal antibodies established by the 1970s pathologists were provided with a significant opportunity to investigate cell lineage and differentiation with neoplastic and non-neoplastic conditions [3]. Diagnostic applications were first realized in hematopathology, especially in particular, lymphomas, but have expanded to all organ systems [4]. The use of IHC to differentiate between tumors that are morphologically indistinct (e.g., hepatocellular carcinoma from metastatic adenocarcinoma in the liver) has become essential [5]. Additionally, the growing use of tissue microarrays and automation has enhanced the reliability/reproducibility and standardization of IHC assays [6]. In the case of breast carcinoma, IHC markers such as THE estrogen receptor (ER), progesterone receptor (PR) and HER2/neu have changed the way treatment is planned [7]. In the same way, IHC is also critical to differentiate lineage-specific markers like TTF-1 for lung origin or PSA for prostatic origin [8]. Additionally, IHC is also critical for the evaluation of prognostic and predictive markers. This includes Ki-67 for proliferation, p53 for tumor suppressor status and PD-L1 for immunotherapy eligibility [9]. More recently, advancements such as multiplex IHC and the utilization of digital pathology integration have further enhanced IHC in diagnosing disease state by enabling the simultaneous detection of various markers [10]. IHC embraces an expanding role in the pathologist's toolkit and remains critical to uniting histomorphology and molecular pathology [11]. Therefore, it is of interest to highlight the expanding diagnostic, prognostic and predictive applications of immunohistochemistry, emphasizing its indispensable role in bridging morphology with molecular pathology and guiding personalized patient care.

## Materials and Methods:

This study was conducted on a retrospective dataset of 200 tissue biopsies received over a 12-month period in a tertiary care hospital. Formalin-fixed, paraffin-embedded (FFPE) tissue samples were subjected to Hematoxylin and Eosin staining initially, followed by IHC staining based on diagnostic needs. Monoclonal antibodies used included CK7, CK20, TTF-1, ER, PR, HER2, CD3, CD20, Ki-67 and p53. IHC was performed using the standard HRP-polymer technique on automated staining platforms. Slides were reviewed independently by two pathologists and discrepancies were resolved through consensus. Statistical analysis was performed using SPSS software.

## Results:

Out of 200 biopsy cases analyzed, immunohistochemistry (IHC) significantly aided in establishing a definitive diagnosis in 160 cases (80%). The most frequent indication for IHC was subtyping of poorly differentiated tumors (45%), followed by confirmation of the primary site in metastatic tumors (30%). ER/PR positivity was detected in 68% of breast carcinoma cases and HER2 positivity in 55%. High Ki-67 index (>20%) was observed in 54% of aggressive lymphomas. TTF-1 showed 85% positivity in primary lung adenocarcinomas, while PSA was positive in 90% of prostatic adenocarcinomas. Discrepant cases (5%) arose from poor antigen retrieval, inadequate fixation, or observer discordance. Overall, IHC proved invaluable in enhancing diagnostic accuracy, tumor classification and therapeutic guidance. Table 1 (see PDF) confirmed that IHC significantly enhanced diagnostic yield across tumor types, especially in breast and prostatic carcinoma. Table 2 (see PDF) showed the most frequently applied markers, highlighting Ki-67, ER/PR and TTF-1 as central to diagnostic workups. Table 3 (see PDF) demonstrated the role of IHC in tumor subtyping, improving classification of breast, lung, lymphomas and metastatic carcinomas. Table 4 (see PDF) detailed common interpretive and technical challenges, while Table 5 (see PDF) outlined discrepant cases and their causes. Collectively, the findings underscore the crucial role of IHC in diagnostic pathology, providing accuracy and therapeutic guidance despite some limitations.

## Discussion:

IHC has emerged as a critical adjunct in diagnostic histopathology, especially in resolving diagnostically challenging cases [1]. In breast pathology, the expression of ER, PR and HER2 is not only diagnostic but also prognostic and predictive for targeted therapy [7]. TTF-1 is a nuclear marker that distinguishes pulmonary adenocarcinomas from other metastatic tumors with high sensitivity and specificity [8]. Similarly, Ki-67 index is routinely employed to evaluate tumor proliferation, influencing decisions in both lymphoma and breast cancer management [9]. Immunohistochemistry (IHC) is a useful tool in identifying tumor lineage with the use of cytokeratin panels (CK7/CK20) facilitating a more straightforward metastatic adenocarcinoma workup [12]. It also contributes to classification of lymphomas with use of C-cell and T-cell markers (i.e. CD20 for B-cells, CD3 for T-cells), which affect the choice of treatment regimen [13]. Furthermore, p53 and PD-L1 evaluation is involved with prognosis and if a patient would be eligible immunotherapy respectively, highlighting the growing role scope of IHC in pathology [10]. IHC does come with faces limitations including variable fixation, antigen retrieval challenges and the subjective nature of interpretation of the staining of a specimen [14]. Automation and digital pathology can help minimize these types of limitations through increased reproducibility and quantitative measurements [15]. Recent trends in multiplex IHC especially with regards to artificial intelligence-based image analysis will make assessment of several biomarkers easier and more accurate in the future [16]. Moreover, new markers such as INSM1 for neuroendocrine tumors and BRG1 for undifferentiated tumors assist with diagnostic evaluation [17]. IHC is a helpful modality in identifying infections such as cytomegalovirus (CMV), herpes simplex virus (HSV) and Helicobacter pylori if the morphology is unclear [18]. IHC is the link between morphology and molecular pathology, making it an essential component of modern pathology practice [19, 20].

## Conclusion:

Immunohistochemistry enhances diagnostic accuracy in pathology by identifying tumor types, subtypes and therapeutic targets. Despite challenges, its evolving applications continue to expand in diagnostic use. Future advances aim to integrate IHC with molecular tools for personalized treatment.
